# Association of Photopic and Mesopic Contrast Sensitivity in older drivers with risk of motor vehicle collision using naturalistic driving data

**DOI:** 10.1186/s12886-020-1331-7

**Published:** 2020-02-04

**Authors:** Cynthia Owsley, Thomas Swain, Rong Liu, Gerald McGwin, Mi Young Kwon

**Affiliations:** 1 Department of Ophthalmology and Visual Sciences, School of Medicine, University of Alabama at Birmingham, 1720 University Blvd., Suite 609, Birmingham, AL 35294-0009 USA; 2 Department of Epidemiology, School of Public Health, University of Alabama at Birmingham, 1665 University Blvd, Birmingham, AL 35294-0022 USA

**Keywords:** Driver safety, Contrast sensitivity, Aging

## Abstract

**Background:**

Older drivers have a crash rate nearly equal to that of young drivers whose crash rate is the highest among all age groups. Contrast sensitivity impairment is common in older adults. The purpose of this study is to examine whether parameters from the photopic and mesopic contrast sensitivity functions (CSF) are associated with incident motor vehicle crash involvement by older drivers.

**Methods:**

This study utilized data from older drivers (ages ≥60 years) who participated in the Strategic Highway Research Program Naturalistic Driving Study, a prospective, population-based study. At baseline participants underwent photopic and mesopic contrast sensitivity testing for targets from 1.5–18 cycles per degree. Model fitting generated area under the log CSF (AULCSF) and peak log sensitivity. Participant vehicles were instrumented with sensors that captured continuous driving data when the vehicle was operating (accelerometers, global positioning system, forward radar, 4-channel video). They participated for 1–2 years. Crashes were coded from the video and other data streams by trained analysts.

**Results:**

The photopic analysis was based on 844 drivers, and the mesopic on 854 drivers. Photopic AULCSF and peak log contrast sensitivity were not associated with crash rate, whether defined as all crashes or at-fault crashes only (all *p* > 0.05). Mesopic AULCSF and peak log sensitivity were associated with an increased crash rate when considered for all crashes (rate ratio (RR): 1.36, 95% CI: 1.06–1.72; RR: 1.28, 95% CI: 1.01–1.63, respectively) and at-fault crashes only (RR: 1.50, 95% CI: 1.16–1.93; RR: 1.38, 95% CI: 1.07–1.78, respectively).

**Conclusions:**

Results suggest that photopic contrast sensitivity testing may not help us understand future crash risk at the older-driver population level. Results highlight a previously unappreciated association between older adults’ mesopic contrast sensitivity deficits and crash involvement regardless of the time of day. Given the wide variability of light levels encountered in both day and night driving, mesopic vision tests, with their reliance on both cone and rod vision, may be a more comprehensive assessment of the visual system’s ability to process the roadway environment.

## Background

Older drivers are the fastest growing group of drivers in the United States (US) [1], both in terms of the number of drivers and the number of annual miles driven. They have a crash rate nearly equal to that of young drivers whose crash rate is the highest among all age groups [2, 3]. Research over the past several decades has indicated that vision impairment contributes to older drivers’ increased crash risk [4–6]. The vast majority of studies on vision and older driver safety have utilized accident reports from police agencies as the outcome measure for collision involvement. In the Unites States these accident reports are routinely indexed by the state jurisdiction in which they occur, with many states making these reports available to scientists (after appropriate regulatory approval) for the purposes of traffic safety research. While these reports provide a wealth of information about the circumstances of a crash (e.g., driver age, place, weather conditions, vehicles involved), they reveal little to nothing about visual and other mechanisms underlying the occurrence of a crash. An alternative to using accident reports to study risk factors for crash involvement is to conduct naturalistic driving studies [7, 8]. Naturalistic driving data are generated by participants driving their own vehicles in the course of their everyday life over long observation periods (e.g., one to two years). Their vehicles are unobtrusively equipped with sensors and video cameras, which record vehicle kinematics, global positioning system (GPS) location, presence of near-by objects, driver behavior (e.g., gaze direction, secondary task activity), and the roadway environment. Naturalistic data provide an unprecedented level of objective detail on safety critical events such as crashes including pre-crash information about driver behavior and roadway contextual factors.

It is well established that spatial contrast sensitivity impairment is common among older adults [9–12]. As with most of the vision and driver safety research literature, studies on contrast sensitivity and crash risk have relied on accident reports comparing collision rates for those with contrast sensitivity impairment to those with no or minimal impairment. While some studies report that contrast sensitivity impairment is associated with a recent history of crash involvement [13, 14] others have reported no association between contrast sensitivity and incident (or future) crash involvement [15–17]. Contrast sensitivity impairment from age-related cataract is associated with a history of higher collision rates [18] and on-road driving performance problems [19, 20]. Drivers with contrast sensitivity impairment secondary to Parkinson’s disease experience on-road difficulties [21–24]. Impaired contrast sensitivity regardless of etiology has also been associated with on-road driving problems [25, 26].

Older driver studies on contrast sensitivity and collision involvement up until now have for the most part assessed contrast sensitivity using a letter chart, specifically the Pelli-Robson chart [27]. The Pelli-Robson chart measures how much contrast is required to identify letters subtending 2.8° of visual angle; letter size is not varied on the chart, but letter contrast is. The chart is a popular choice in epidemiological and clinical studies because it is brief, easy to administer, and has good reproducibility [28–30]. It also has confirmed construct validity for everyday visual task performance in that reduced contrast sensitivity as measured by the chart is associated with adverse outcomes such as falls [31], mortality [32], performance mobility deficits [33], and slowed reading [34]. A limitation of the Pelli-Robson chart is that it does not measure a person’s contrast sensitivity as a function of spatial frequency (i.e., target size) [35].

Prior research using SHRP2 data from older drivers [36] examined the relationship between contrast sensitivity and crash risk. In SHRP2 contrast sensitivity was assessed by the Optec 6500 P which measures contrast sensitivity for five spatial frequency targets ranging from 1.5 to 18 cycles per degree (cpd) [37]. Contrast sensitivity results were presented for some but not all spatial frequencies tested, with no explanation provided for why some were omitted. It appears that only statistically significant results were presented.

When a set of thresholds for a range of spatial frequencies is measured as done in SHRP2, a model can be used to fit thresholds to form a spatial contrast sensitivity function (CSF); many CSF models have been put forth [38, 39]. Some of these models can provide good fits to the raw data with only 4 parameters [40]. The major advantage of measuring the CSF, rather than contrast sensitivity for a single target size, is that it constitutes a comprehensive summary of visibility for a broad variety of spatial stimuli [39].

The purpose of this study is to examine the association of photopic and mesopic CSFs with motor vehicle crash involvement by older drivers using naturalistic driving techniques. Photopic vision is mediated by cone photoreceptors only, whereas mesopic vision is mediated by both cone and rod photoreceptors.

## Methods

### Data source

The study utilized data from the Strategic Highway Research Program (SHRP2) Naturalistic Driving Study, the largest naturalistic driving study conducted to date [8]. Details of the SHRP2 study design and methods have been previously published [41–43]. The study consisted of a large sample of drivers from six study sites in the United States (Bloomington, IN; State College, PA; Tampa Bay, FL; Buffalo, NY; Durham, NC; Seattle, WA), representing various geographies, climates, state laws, and road types. A combination of random-digit dialing and public advertising was used to recruit more than 18,000 individuals for screening [41]. The Virginia Tech Center for Survey Research phoned potential participants to discuss the study protocol and confirm eligibility. Individuals who were licensed drivers, drove at least 3 days per week, planned to keep the vehicle for the duration of the study, and had an eligible and mechanically sound vehicle were eligible to participate [41]. Participants completed a standard intake process during a single in-person visit at regional sites. Participants were enrolled from October 2010 through December 2013 and most were followed for 1 or 2 years. The final SHRP2 sample included 3541 drivers aged 16–98 years. The focus in the present analysis was on the ≥60-year-old subsample of SHRP2 which included 1019 participants, since deficits in spatial contrast sensitivity typically become more prevalent by the 60s [9–12]. The Institutional Review Board (IRB) of the National Academy of Sciences, Virginia Tech Transportation Institute (VTTI), and the regional study sites provided oversight for the SHRP2 study [44]. The analysis reported here was approved by the IRB of the University of Alabama at Birmingham. The study followed the tenets of the Declaration of Helsinki.

At the enrollment visit, participants completed a questionnaire on demographic characteristics (age, gender, race/ethnicity). Contrast sensitivity was measured for each eye separately using habitual correction under both daytime conditions (85 cd/m^2^, photopic condition) and night-time conditions (3 cd/m^2^, mesopic condition) using the Optec 6500 P (Stereo Optical Co., Inc., Chicago, IL), a contrast sensitivity screening device [37]. It is based on the Functional Acuity Contrast Test [45]. The test presents sine-wave grating stimuli of 1.5, 3, 6, 12, and 18 cpd as nine circular patches, with each patch being 1.5 log contrast decreased from the one before it. The grating pattern can be oriented to the left, up, or right, creating a three-alternative forced-choice response format. The participant is asked to indicate the orientation of the grating. Testing for a given spatial frequency is terminated at the first incorrect response, with the last correct response designated as the contrast sensitivity for that spatial frequency.

### CSF model fitting

**Fig. 1:**
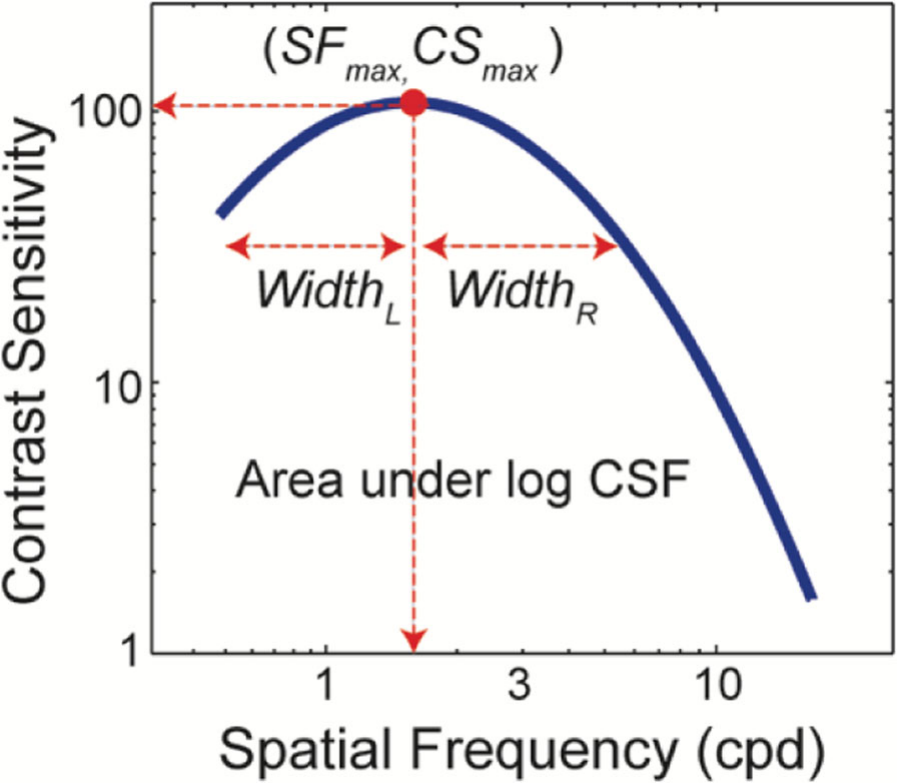
Illustration of the contrast sensitivity function (CSF). The spatial contrast sensitivity function, defined as the reciprocal of the contrast threshold as a function of spatial frequency, can be described by four parameters: the peak contrast sensitivity, *CS*_*max*_; the peak spatial frequency, *SF*_*max*_; degree of curvature of the left and right branches of the asymmetric parabolic function, *width*_*L*_ and *width*_*R*_. In addition, we can obtain a summary statistic of contrast sensitivity: area under log CSF (AULCSF) which represents the overall contrast sensitivity of the function

The contrast sensitivity data (i.e., 1/contrast threshold versus spatial frequency) were fitted with an asymmetric parabolic function shown to well describe both normal and low vision CSFs [46]. The function has four parameters as shown by the following equation:

$$ f(SF)=\left\{\begin{array}{c}\ {CS}_{max}-{\left( SF-{SF}_{max}\right)}^2\times {\left({\mathrm{width}}_L\right)}^2\  if\  SF<{SF}_{max}\\ {}{CS}_{max}-{\left( SF-{SF}_{max}\right)}^2\times {\left({\mathrm{width}}_R\right)}^2\  if\  SF\ge {SF}_{max}\end{array}\right., $$

where *f* (*SF*) is the contrast sensitivity at a spatial frequency *SF*, *CS*_*max*_ is the peak contrast sensitivity, *SF*_*max*_ is the peak spatial frequency, the spatial frequency at which *CS*_*max*_ occurs, and *width*_*L*_ and *width*_*R*_ are the left and right bandwidths of the asymmetric parabolic function, respectively (Fig. 1). The values for contrast sensitivity and spatial frequency were log-transformed before curve-fitting. The fits were achieved using a simplex search method [47] to search for the optimal fit producing the least squares error. The goodness of the fit was evaluated with *R* squared for each individual curve-fit.

As demonstrated by Chung and Legge [46], it is reasonable to fit low-vision CSF data with the asymmetric parabolic function with fixed *Width*_*L*_ and *Width*_*R*_ parameter values that are obtained from normal-vision CSFs. In other words, the asymmetric parabolic function with two free parameters and two fixed *Width* parameter values are sufficient enough to describe the CSF data under different visual conditions. In this study, we, thus, adopted the “*template fit”* approach used by Chung and Legge [46]. We adopted *Width*_*L*_ and *Width*_*R*_ parameter values estimated from the fitted curves of our 70 best photopic CSF data. By reducing the number of free parameters, we were able to avoid overfitting that might have occurred in data from a subset of subjects. We, however, confirmed that this *template-fit* approach yields a reasonable fit to the CSF data across subjects and viewing conditions by evaluating the goodness of the fit with *R*^2^ for each individual curve-fit. More than 80% of individual curve-fits showed a *R*^2^ value greater than 0.8 and more than 90% of individual fits showed a *R*^2^ value greater than 0.5. Participants with an *R*^2^ less than 0.5 were removed from the study. To relate the CSF to driving performance, we examined the peak contrast sensitivity, *CS*_*max*_ and area under the log CSF (AULCSF) which represents the overall contrast sensitivity of the subject. Driving uses both eyes, therefore binocular contrast sensitivity measures were created. Since SHRP2 did not test the CSF under binocular viewing conditions, the binocular CSF for each subject was constructed by using the better contrast sensitivity of the two eyes for a given spatial frequency.

### Naturalistic driving data

While the participant underwent the enrollment visit, the personal vehicle of each participant was instrumented with a data acquisition system (DAS) that captured continuous driving data anytime the vehicle was operating. Participants were instructed to drive their vehicles as they normally would while enrolled in the study; enrollees participated for either one or 2 years. Results from other naturalistic driving studies demonstrate that participants adapt quickly to the instrumented vehicle, engaging in generally typical driving activities and reporting they are not unduly influenced by the equipment installed in their vehicles [48, 49]. The DAS included several types of sensors including accelerometers, global positioning system (GPS), forward radar, a color video camera view of the forward roadway, and three grayscale video cameras of the rear view, the driver’s face, and view over the driver’s right shoulder.

At regular intervals, data were transmitted to VTTI for processing. After removing trip files from non-consenting drivers (i.e., persons other than the participant who drove the vehicle), trained VTTI analysts reviewed video data when vehicle physical sensors detected [1] large changes in speed or position of the car with respect to the road, [2] activation of advanced safety systems (e.g. anti-lock braking), [3] the participant pushed the critical incident button to flag an event, or [4] the analysts detected an event [50]. A short window of video surrounding the potential event was reviewed to verify and classify the event as a crash or near-crash [51]. Crashes were defined as events where the SHRP2 participant’s vehicle made contact with any object (vehicles, pedestrians, cyclists, animals, tree, buildings), at any speed, including non-premeditated departures from the roadway where at least one tire left the paved or intended travel surface of the road. At-fault crash status of the driver was determined by VTTI analysts and was only coded if there was observable evidence in the video that the driver committed an error that led to the crash. VTTI analysts coding crash events were masked to each participant’s status on variables collected at the enrollment visit. Inter-rater agreement on classifying events was periodically assessed for VTTI analysts and compared to an expert rater, with the overall agreement being 88% for crash events [52]. The number of miles driven during the participant’s enrollment period was based on the odometer reading or, if unavailable, the GPS data.

### Statistical analysis

Demographic characteristics and contrast sensitivity parameters (AULCSF and peak contrast sensitivity) were summarized for the sample. Impaired contrast sensitivity parameters were defined by those values in the lowest quartile of the distribution for AULCSF and peak contrast sensitivity. Poisson regression was utilized to model age adjusted rate ratios (RR) and 95% confidence intervals (95% CI) for AULCSF and peak contrast sensitivity for all crashes and all at-fault crashes regardless of the time of day of the crash. Models were fit using a log-link, log of miles driven as the offset, and the level of significance set to < 0.05.

## Results

Of the 1019 participants in SHRP2 ≥ 60 years old, 89 were missing both photopic or mesopic contrast sensitivity data, resulting in 930 persons available for analysis. When fitting photopic CSF models, an additional 9 were missing photopic data, and 77 had *R*^*2*^ < 0.5 for the model fit, and thus were deleted, resulting in a total of 844 persons with photopic contrast sensitivity parameters. When fitting mesopic CSF models, an additional 7 were missing mesopic data and 69 had *R*^*2*^ < 0.5 for model fit and thus were deleted, resulting in 854 with mesopic contrast sensitivity parameters.

Approximately ¾ of the sample were in their 60s or 70s with the remaining ≥80 years old (Table 1). There were slightly more men than woman. The vast majority of participants (over 95%) were white of non-Hispanic origin. Table 2 shows the mean and standard deviation of contrast sensitivity parameters for the sample. Consistent with the literature [53], mesopic AULCSF and peak sensitivity were lower than those parameters for photopic contrast sensitivity.

**Table 1:** Demographic characteristics of the sample (*N* = 915)

Characteristic	n	% ^a^
Age, years		
60–69	310	33.9
70–79	396	43.3
80–90	200	21.9
90–99	9	1.0
Gender		
Men	491	53.7
Women	424	46.3
Race		
White	871	95.2
Black	15	1.6
Other^b^	22	2.4
Unknown	7	0.8
Ethnicity		
Hispanic or Latino	12	1.3
Not Hispanic or Latino	872	95.3
Unknown	31	3.4

^a^Percents may not sum to 100 due to rounding

^b^Other includes Asian, American Indian or Alaska Native, and Native Hawaiian or Other Pacific Islander

**Table 2:** Contrast sensitivity parameters for the sample

	Mean	Standard Deviation
Photopic contrast sensitivity parameters (*N* = 844)		
AULCSF ^a^	1.70	0.37
Peak log sensitivity	1.89	0.21
Mesopic contrast sensitivity parameters (*N* = 854)		
AULCSF ^a^	1.25	0.37
Peak log sensitivity	1.75	0.23

^a^AULCSF is area under the log contrast sensitivity function

Participants drove a total of 7,417,879 miles with an average of 8107 ± 6967 miles driven over 233 ± 183 h of driving time per person. The most common vehicle driven was a passenger car (68.1%), followed by sport utility vehicle or pickup (17.6%), mini-van (5.8%), crossover (1.9%), and unknown (6.6%). Of the 915 participants, 688 had no crashes, 161 had 1 crash, 44 had 2 crashes, 9 had 3 crashes, 5 had 4 crashes, 2 had 5 crashes, 1 had 6 crashes, 3 had 7 crashes, 1 had 8 crashes, and 1 had 13 crashes. This resulted in 354 total crash events among 227 participants. Of the 354 crashes, 307 were deemed at-fault for the participant driving. The majority of crashes (272 of 354, 77%) and at-fault crashes (237 of 307, 77%) occurred during the daytime.

Table 3 lists the age-adjusted associations between photopic and mesopic contrast sensitivity parameters and crash rate. Photopic AULCSF and peak log contrast sensitivity were not associated with crash rate, whether defined as all crashes or at-fault crashes only (all *p* > 0.05). Mesopic AULCSF was associated with an increased crash rate when defined in terms of all crashes; drivers in the lowest AULCSF quartile were 36% more likely to incur crashes per mile driven than were those in the upper three quartiles (RR: 1.36, 95% CI: 1.06–1.72). The association between peak mesopic log sensitivity and crash involvement was slightly weaker than that for AULCSF but statistically significant, with drivers in the lowest log sensitivity quartile 28% more likely to be crash involved (RR: 1.28, 95% CI: 1.01–1.63). Both associations were stronger when considering only at-fault crashes; drivers in the lowest AULCSF and peak log sensitivity quartiles were 50 and 38% more likely, respectively, to be crash involved as compared to those in the upper three quartiles (RR: 1.50, 95% CI: 1.16–1.93; RR: 1.38, 95% CI: 1.07–1.78).

**Table 3:** Age-adjusted associations between impaired contrast sensitivity and rate of motor vehicle crash involvement for all crashes and for at-fault crashes

	All Crashes (N events = 354)		At-fault Crashes (N events = 307)	
	Rate ratio (95% confidence interval)	*P*-value	Rate ratio (95% confidence interval)	*P*-value
Photopic contrast sensitivity parameters				
AULCSF^a^	0.80 (0.61–1.05)	0.102	0.77 (0.57–1.03)	0.077
Peak log sensitivity	0.80 (0.61–1.04)	0.091	0.77 (0.58–1.03)	0.072
Mesopic contrast sensitivity parameters				
AULCSF^a^	1.36 (1.06–1.72)	0.015	1.50 (1.16–1.93)	0.002
Peak log sensitivity	1.28 (1.01–1.63)	0.042	1.38 (1.07–1.78)	0.014

^a^AULCSF is area under the log contrast sensitivity function

## Discussion

In a prospective population-based study on older drivers in the US, impaired photopic contrast sensitivity was not associated with an increased rate for incident motor vehicle crash involvement. Our results agree with two previous prospective, population-based studies on older drivers, also performed in the US [16, 17]. It is the case that other studies have found positive associations between photopic contrast sensitivity deficits and crash involvement but the outcome variable in these studies was a history of crashes, not future crashes [13, 14]. The limitation with using study designs where the outcome is crashes retrospective to enrollment is that one does not know whether, for example, the photopic contrast sensitivity impairment existed before the crash. These studies taken together provide little support for photopic contrast sensitivity testing at the population level for the purposes of reducing the burden of motor vehicle crashes.

It is useful to consider why photopic contrast sensitivity deficit is unrelated to future crash risk. First, studies have shown that contrast sensitivity impairment is related to self-reported driving difficulty [54] as well as self-regulation [55]. Driver’s with photopic contrast sensitivity deficits are more likely to avoid challenging driving situations [55, 56], reduce driving exposure (i.e., mileage) [55], and are at greater risk for driving cessation [57, 58]. Second, one of the most common causes of contrast sensitivity loss is age-related cataract. However, most older adults in the US undergo cataract surgery and intraocular lens implantation after cataracts start hampering their visual daily activities. Cataract surgery reduces motor vehicle crash risk by 50% [59]. Thus, while contrast sensitivity loss has a negative impact on driving habits, its potential negative impact on crash risk appears to be mitigated by changes in driving habits and the wide-spread availability of cataract surgery.

Although the association between photopic contrast sensitivity and crash involvement was null in our study, mesopic contrast sensitivity impairment was associated with incident crashes and at-fault crashes. For example, drivers with impaired mesopic sensitivity as defined by AULCSF were 50% more likely to incur an at-fault crash as compared to those who were unimpaired. That mesopic contrast sensitivity impairment elevates collision risk in older drivers is a novel finding for the literature. Previous studies suggest that mesopic vision deficits contribute to driver safety and performance problems in studies of samples of drivers that have a wide range of ages, but not specifically focused on older drivers. Lachenmayr et al. [60] reported that drivers with worse mesopic vision were more likely to be involved in night-time collisions, however 2/3 of those studied were not older adults, but were ≤ 60 years old. A study on bus and truck drivers found that those with “reduced twilight vision” were more frequently involved in collisions [61]. Older drivers with mesopic vision acuity impairment exhibited worse night-time driving performance on a closed driving course [62]. Black et al. found that correcting astigmatism in young drivers with toric contact lenses improved on-road driving performance which was also linked to better mesopic vision [63].

A strength of this study is the use of incident crashes as an outcome measure based on naturalistic driving recordings. The study was population-based and included a large sample of older drivers. This is the first study to utilize a contrast sensitivity function model and the parameters it generates to examine associations between older driver safety and contrast sensitivity. A strength of this approach is that it comprehensively considers the entire spatial envelope of visibility, not each spatial target’s piecemeal role in collision risk (the approach used in a previous report using SHRP2 data [36]). In that report [36], the association between collision involvement and contrast sensitivity was evaluated separately at each of 4 spatial frequencies under photopic and mesopic viewing conditions; that is, they did not fit a CSF model to the data before analysis. A limitation of our study is an insufficient sample size and number of crash events in order to study contrast sensitivity and crash involvement stratified by day- versus night-time crashes.

## Conclusion

This study agrees with previous research [16, 17] that photopic contrast sensitivity testing is not useful for understanding future crash risk at the older-driver population level. Although many studies have linked photopic contrast sensitivity impairment to a history of crash involvement, the preponderance of evidence thus far suggests that photopic contrast sensitivity will be an ineffective screening tool to identify older drivers who are likely to be crash involved in the future. This study highlights a previously unappreciated association between older adults’ mesopic contrast sensitivity deficits and crash involvement regardless of the time of day. Mesopic vision is mediated by both cone and rod photoreceptors. Given the wide variability of light levels encountered in both day and night driving and the many environmental features impacting roadway illumination, mesopic vision tests, with their reliance on both cone and rod vision, may be a more comprehensive assessment of the visual system’s ability to process the roadway environment.

## Data Availability

Data from the SHRP2 study are available through the Virginia Tech Transportation Institute. Information on data access is provided at https://insight.shrp2nds.us.

## Abbreviations

AULCSF: Area under log contrast sensitivity function
CSF: Contrast sensitivity function
CS_max_: Contrast sensitivity_max_
DAS: Data acquisition system
GPS: Global positioning system
IRB: Institutional Review Board
RR: Rate ratio
SF: Spatial frequency
SHRP2: Strategic Highway Research Program
US: United States
